# Combining Clinical-Radiomics Features With Machine Learning Methods for Building Models to Predict Postoperative Recurrence in Patients With Chronic Subdural Hematoma: Retrospective Cohort Study

**DOI:** 10.2196/54944

**Published:** 2024-08-28

**Authors:** Cheng Fang, Xiao Ji, Yifeng Pan, Guanchao Xie, Hongsheng Zhang, Sai Li, Jinghai Wan

**Affiliations:** 1 Department of Neurosurgery National Cancer Center/National Clinical Research Center for Cancer/Cancer Hospital Chinese Academy of Medical Sciences and Peking Union Medical College Beijing China; 2 Department of Neurosurgery The First Affiliated Hospital of Anhui Medical University Anhui Public Health Clinical Center Hefei China; 3 The School of Big Data and Artificial Intelligence Anhui Xinhua University Hefei China; 4 Department of Neurosurgery The Second Affiliated Hospital of Anhui Medical University Anhui Medical University Hefei China

**Keywords:** chronic subdural hematoma, convolutional neural network, machine learning, neurosurgery, radiomics, support vector machine

## Abstract

**Background:**

Chronic subdural hematoma (CSDH) represents a prevalent medical condition, posing substantial challenges in postoperative management due to risks of recurrence. Such recurrences not only cause physical suffering to the patient but also add to the financial burden on the family and the health care system. Currently, prognosis determination largely depends on clinician expertise, revealing a dearth of precise prediction models in clinical settings.

**Objective:**

This study aims to use machine learning (ML) techniques for the construction of predictive models to assess the likelihood of CSDH recurrence after surgery, which leads to greater benefits for patients and the health care system.

**Methods:**

Data from 133 patients were amassed and partitioned into a training set (n=93) and a test set (n=40). Radiomics features were extracted from preoperative cranial computed tomography scans using 3D Slicer software. These features, in conjunction with clinical data and composite clinical-radiomics features, served as input variables for model development. Four distinct ML algorithms were used to build predictive models, and their performance was rigorously evaluated via accuracy, area under the curve (AUC), and recall metrics. The optimal model was identified, followed by recursive feature elimination for feature selection, leading to enhanced predictive efficacy. External validation was conducted using data sets from additional health care facilities.

**Results:**

Following rigorous experimental analysis, the support vector machine model, predicated on clinical-radiomics features, emerged as the most efficacious for predicting postoperative recurrence in patients with CSDH. Subsequent to feature selection, key variables exerting significant impact on the model were incorporated as the input set, thereby augmenting its predictive accuracy. The model demonstrated robust performance, with metrics including accuracy of 92.72%, AUC of 91.34%, and recall of 93.16%. External validation further substantiated its effectiveness, yielding an accuracy of 90.32%, AUC of 91.32%, and recall of 88.37%, affirming its clinical applicability.

**Conclusions:**

This study substantiates the feasibility and clinical relevance of an ML-based predictive model, using clinical-radiomics features, for relatively accurate prognostication of postoperative recurrence in patients with CSDH. If the model is integrated into clinical practice, it will be of great significance in enhancing the quality and efficiency of clinical decision-making processes, which can improve the accuracy of diagnosis and treatment, reduce unnecessary tests and surgeries, and reduce the waste of medical resources.

## Introduction

### Background

Chronic subdural hematoma (CSDH) is a prevalent neurosurgical pathology, disproportionately affecting middle-aged and older adults. Epidemiological data indicate incidence rates of 13.5/100,000, escalating to 58.1/100,000 in individuals aged ≥65 years [1,2]. Manifestations commonly include headache, nausea, vomiting, and diplopia, indicative of elevated intracranial pressure. Diagnosis is generally confirmed through cranial computed tomography (CT) or magnetic resonance imaging. Established as a medical condition since 1857, surgical intervention remains a proven, efficacious treatment modality for CSDH. However, postoperative recurrence serves as a critical metric for evaluating surgical success [3]. Such recurrence imposes not only physical suffering on patients but also accentuates financial burden on families and health care systems. For patients who are older, have a history of multiple surgeries, or have other complications, an accurate predictive tool can help physicians identify these high-risk patients in advance, allowing for a more precise treatment and follow-up plan. Each patient with CSDH has a different condition and clinical background, and thus requires a personalized treatment plan that can assess the risk of postoperative recurrence based on the patient’s specific clinical information (age, sex, medical history, symptoms, signs, and imaging manifestations). Given the increasing strain on medical resources, optimizing the allocation and use of medical resources and improving the operational efficiency of hospitals has become an important issue. Hence, in the above clinical context, the development of a predictive tool for postoperative recurrence risk is integral for informed clinical decision-making and optimized treatment outcomes, which can bring greater benefits to both patients and the health care system.

Recent advancements in computer technology have facilitated the construction of predictive models anchored on clinically pertinent data. Machine learning (ML) has emerged as a particularly robust paradigm, capable of delineating complex, nonlinear relationships between variables and outcomes. A plethora of studies substantiate the impressive levels of accuracy and reliability achieved through ML applications [4-7]. This study uses 4 ML methodologies—convolutional neural networks (CNNs), support vector machines (SVMs), random forest (RF), and linear regression (LR)—each enjoying widespread academic acceptance and demonstrated applicability in predictive research across various domains, including health care and food sciences [6,8,9].

By surveying existing literature, it is evident that ML models integrated with radiomics are garnering increased scholarly attention [10,11]. Radiomics constitutes a novel approach in medical image analysis, principally centered on quantitative feature extraction. This technique transforms medical imagery into high-dimensional structures, facilitating the comprehensive analysis of regions of interest (ROIs) in conjunction with relevant clinical, diagnostic, and prognostic data. A typical radiomics workflow encompasses stages of image acquisition, reconstruction, preprocessing and processing, feature extraction, selection, and eventually, classification or regression modeling [12]. Although previous research has melded radiomics and ML for diagnostic and prognostic applications in other medical specialties such as dermatology, oncology, and cardiology [13-15], studies targeting CSDH remain comparatively scant.

### Objective

The objective of this investigation is to amalgamate ML algorithms with radiomics and clinical variables for the construction of a predictive model aimed at gauging the risk of CSDH recurrence after surgery. The study will rigorously compare various methodologies and models to identify the most efficacious predictive framework for CSDH recurrence.

## Methods

### Ethical Considerations

The retrospective study was approved (approval no PJ-YX2024-021) by the Ethics Committee of the First Affiliated Hospital of Anhui Medical University (Anhui Public Health Clinical Center).

### Participants

**Figure 1:**
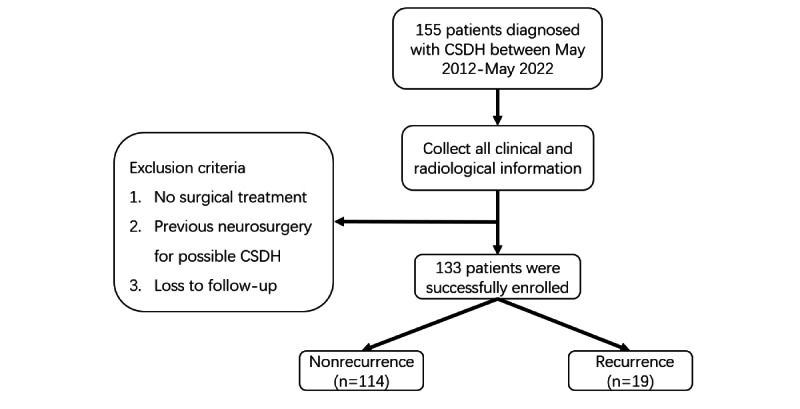
Patient selection and enrollment process. CSDH: chronic subdural hematoma.

We compiled clinical and radiological data from patients diagnosed with CSDH who were treated at the neurosurgery department of the Second Affiliated Hospital of Anhui Medical University between May 2012 and May 2022. The inclusion criteria were as follows: (1) confirmed clinical diagnosis of CSDH; (2) participants who underwent surgical intervention, either Burr hole craniostomy or craniotomy; and (3) availability of comprehensive clinical records, encompassing treatment histories, preoperative and postoperative imaging examinations, laboratory analyses, among other pertinent data. Exclusion criteria included the following: (1) patients who exhibited symptomatic improvement via pharmacological intervention, obviating the need for surgical treatment; (2) any history of prior neurosurgical procedures that could potentially induce CSDH; and (3) cases with incomplete follow-up data or where recurrence after surgery was undetermined. Following these criteria, 133 patients were incorporated into the study, and there are no missing values for all case data in this study. The process of patient selection and enrollment is delineated in Figure 1. Furthermore, an external validation set was generated by screening data from 20 patients with CSDH who underwent treatment at the First Affiliated Hospital of Anhui Medical University.

### Clinical Data

Upon a rigorous review of existing literature and consultations with experienced neurosurgeons, specific data parameters were established for model construction. These parameters were extracted from the electronic case files of the participants and included: patient demographics (age and sex), pertinent clinical history (smoking or drinking habits, prior medical history, history of head trauma, and history of antiplatelet or anticoagulant therapy), preoperative clinical grading based on the Markwalder Grading Scale (MGS), and duration of hospitalization. Imaging data encompassed variables such as the location of the CSDH (unilateral or bilateral), hematoma classification, preoperative hematoma volume, preoperative midline shift, postoperative midline improvement, and cranial CT scans. Subsequent to the follow-up period, 19 patients exhibited postoperative recurrence. These patients were bifurcated into 2 cohorts: those with recurrence and those without recurrence. Clinical indices used in model construction are elaborated in Table 1. Continuous variables were subjected to 2-tailed *t* test analyses and are represented as mean (SD). Categorical variables were analyzed using chi-square test and are conveyed as percentages. Statistical analysis was performed using SPSS Statistics (version 27.0; IBM Corp).

In Textbox 1, we delineate the grading criteria and definitions associated with the MGS [16]. Initially proposed in the 1980s, extensive research has validated the MGS as a robust metric for evaluating postoperative neurological recovery and prognosis in patients with CSDH. Specifically, a grade of 0 indicates normal neurological function, grades 1 to 2 signify good neurological function, and grades 3 to 4 represent poor neurological function.

**Table 1:** Clinical variables used to construct the model.

Variables		Nonrecurrence (n=114)	Recurrence (n=19)	*P* value	
Age (y), mean (SD)		70.1 (9.4)	73.7 (8.3)	.13	
**Sex, n (%)**					.50
	Male	91 (79.8)	17 (89)		
	Female	23 (20.2)	2 (11)		
**Smoking or drinking** **, n (%)**					.12
	Yes	56 (49.1)	13 (68)		
	No	58 (50.9)	6 (32)		
**Hypertension** **, n (%)**					.10
	Yes	43 (37.7)	11 (58)		
	No	71 (62.3)	8 (42)		
**Cerebral infarction, n (%)**					.02
	Yes	11 (9.6)	6 (32)		
	No	103 (90.4)	13 (68)		
**History of head trauma, n (%)**					.34
	Yes	73 (64.0)	10 (53)		
	No	41 (36.0)	9 (47)		
**History of antiplatelet or anticoagulation** **, n (%)**					.003
	Yes	10 (8.8)	7 (37)		
	No	104 (91.2)	12 (63)		
**Markwalder Grading Scale, n (%)**					.004
	0	1 (0.9)	0 (0)		
	1	38 (33.3)	0 (0)		
	2	56 (49.1)	12 (63)		
	3	18 (15.8)	7 (37)		
	4	1 (0.9)	0 (0)		
Length of stay in hospital (d), mean (SD)		14.0 (4.3)	14.5 (3.2)	.64	
**CSDH^a^ location, n (%)**					.01
	Unilateral	104 (91.2)	13 (68)		
	Bilateral	10 (8.8)	6 (32)		
**Classification of hematoma** **（** **%** **）**					.02
	Homogeneous	50 (43.9)	2 (11)		
	Laminar	12 (10.5)	1 (5)		
	Separated	35 (30.7)	8 (42)		
	Trabecular type	17 (14.9)	8 (42)		
Preoperative hematoma volume (mL), mean (SD)		103.4 (25.0)	127.1 (49.3)	.05	
Preoperative midline shift (cm), mean (SD)		1.1 (0.3)	0.9 (0.3)	.01	
Postoperative midline improvement (cm), mean (SD)		0.6 (0.2)	0.4 (0.2)	.01	

^a^CSDH: chronic subdural hematoma.

**Descriptions of the categories of the Markwalder Grading Scale [16].**

**Grading score and description**

- Grade 0: neurologically normal
- Grade 1: alert and orientated: absence of mild symptoms such as headache, or mild neurological deficit such as reflex asymmetry
- Grade 2: drowsy or disorientated, or variable neurological deficit such as hemiparesis
- Grade 3: stuporous, but responding appropriately to noxious stimuli, several focal signs such as hemiplegia
- Grade 4: comatose with absent motor responses to painful stimuli, decerebrate or decorticate posturing

### Image Data

**Figure 2:**
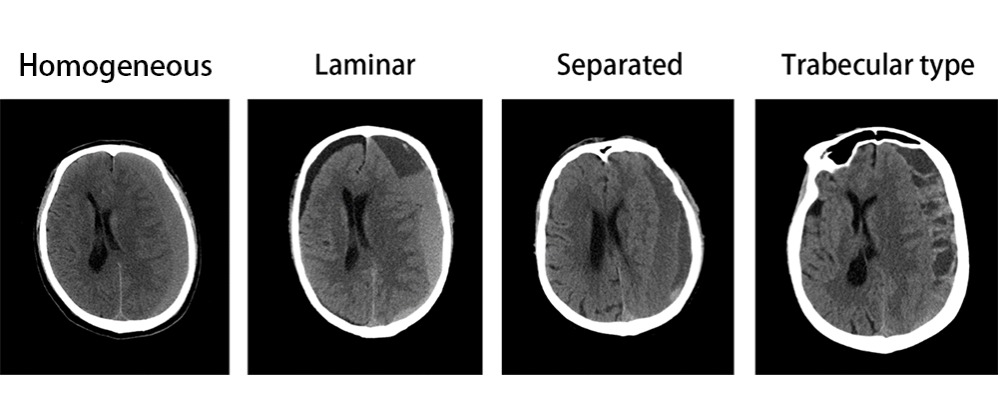
Classification of hematoma.

Complementing the comprehensive clinical data set, preoperative cranial CT scans were acquired for all enrolled participants. Hematoma images were systematically categorized into 4 distinct types: homogeneous, laminar, separated, and trabecular. Representative CT scans for each category are furnished in Figure 2. This taxonomic approach to hematoma classification was initially delineated by Nakaguchi et al [17]. Their work posited that hematomas with more irregular structures correlated with elevated recurrence rates. Subsequently, this classification schema has been integrated into a scoring system aimed at assessing recurrence risk [18]. All procured CT images were stored in DICOM format.

### Radiomics

**Figure 3:**
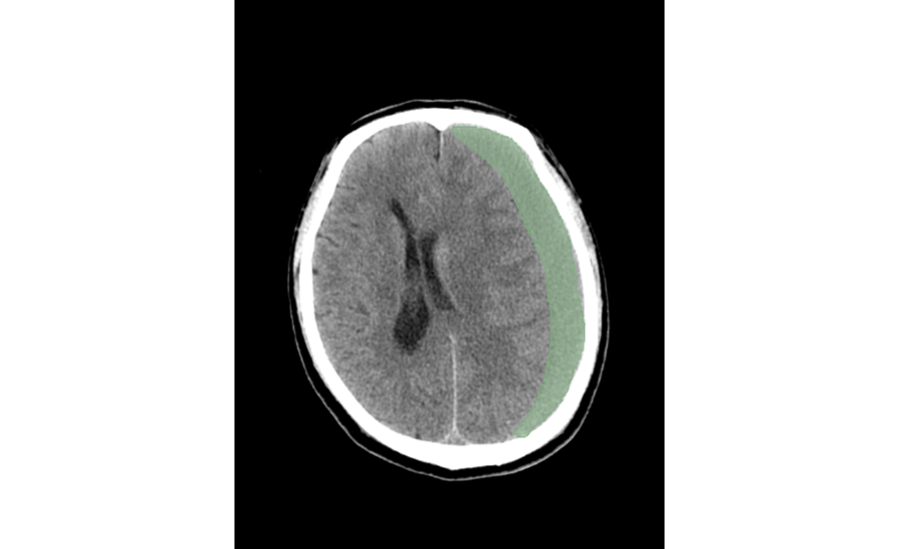
Hand-delineated regions of interest.

Hematoma segmentation was executed through semiautomatic techniques using the 3D Slicer open-source software platform (version 4.10). The ROI, specifically the hematoma, was further segmented using the PyRadiomics package, an open-source plug-in available on the 3D Slicer platform (Figure 3). The use of open-source software enabled the direct computation of 3D features without the necessity for slice-wise combination or averaging. A total of 107 radiomic features were manually extracted from each patient’s CT images. These features were allocated to 7 distinct feature categories: 18 were first-order statistics, 14 were shape based, 24 were derived from gray-level co-occurrence matrices, 16 were derived from gray-level run-length matrices, 16 were derived from gray-level size-zone matrices, 5 were derived from neighboring gray tone difference matrices, and 14 were derived from gray-level dependence matrices. This conversion from image-based to data-driven features optimizes the data set for subsequent computational analyses and research endeavors.

### Modeling

#### Overview

Neuroscientists participated comprehensively in this research, inclusive of data collection and categorical predictive model development. To safeguard patient confidentiality and adhere to the ethical guidelines stipulated by the ethics committee, patient data were anonymized through numerical coding. This approach also ensured the clinical applicability of the study’s findings. We complied with Items of reporting predictive models in biomedical research in this study (Multimedia Appendix 1).

Four ML algorithms were used for model development: CNN, SVM, RF, and LR. Hyperparameter tuning was conducted via a grid search algorithm to optimize model performance. The main components of the CNN structure include an input layer, 3 convolution-pooling layers, 1 flat layer, 2 fully connected layers, and 1 output layer. Textboxes 2 and 3 detail the parameter configurations of the CNN and SVM. The data set was stratified into a training set, comprising 69.9% (93/133) of the samples, and a test set accounting for the remaining 30.1% (40/133). Given the data set’s limited sample size, 5-fold cross-validation was executed on the training set to ensure robustness and validity. This cross-validation technique is a standard practice in ML for its ability to produce reliable performance metrics, mitigate the risks of overfitting and underfitting, and assess the model’s generalization capability. The models were developed using the scikit-learn framework and implemented in the Python 3.9 programming environment.

**Setting hyperparameters for convolutional neural network.**

**Hyperparameter and setting**

- Activation function: rectified linear unit
- Optimizer: Adam
- Batch size: 64
- Dropout: 0.5
- Loss: binary cross-entropy

**Setting hyperparameters for support vector machine.**

**Hyperparameter and setting**

- Kernel: radial basis function
- Gamma: 0.01
- C: 50

#### Input Data Set Selection

Multiple input data set configurations were assessed to optimize the predictive model. Initial models were constructed using 3 distinct types of data: clinical data, CT images, and radiomics from the enrolled patient cohort. These data types were then aggregated in 2 specific combinations to generate models based on mixed input data, specifically, clinical-CT and clinical-radiomics features. Furthermore, a composite model using clinical data, CT images, and histological imaging was also developed. Comparative analysis of these data set configurations was performed to identify the algorithms and input data sets most conducive for predictive modeling.

#### Feature Selection

##### Overview

Our evaluation indicated that the SVM model, when configured with the clinical-radiomics data set, demonstrated superior predictive efficacy. However, the complexity of the input variables compromised both the model’s performance and computational efficiency, thereby not meeting the study’s predefined objectives. To address this, a feature selection strategy was used with the principal aim of isolating the most impactful variables. This was anticipated to enhance model performance, increase computational efficiency, and minimize algorithmic complexity. For this task, the recursive feature elimination (RFE) method was selected, supported by references [19-21]. Specific steps for the RFE implementation will be outlined in the subsequent sections.

##### Initial Feature Subset Evaluation

The composite data set of radiomic and clinical variables serves as the initial feature subset for the SVM model. Each feature’s importance is quantified, and the classification accuracy of this initial feature set is assessed through cross-validation techniques.

##### Iterative Feature Removal and Recalculation

he least impactful feature is excised from the current feature subset, creating a modified feature set. This new set is subsequently input into the SVM model. Feature importance is recalculated and the modified feature subset’s classification accuracy is evaluated using cross-validation methods.

##### Optimizing Feature Selection

The procedure delineated in step 2 is recursively applied until no features remain in the subset. Through this iterative process, a total of 15 distinct feature subsets are generated, each comprising a varying number of features. The feature subset yielding the highest classification accuracy is identified as the optimal feature combination for the predictive model.

##### Clinical Settings and Modeling Background

The clinical settings for our predictive model include the following: (1) Facility type—it is mainly applied to neurosurgery wards in general hospitals or neurosurgery specialty hospitals, which are capable of handling complex neurosurgical procedures. (2) Size—it is more suitable to be implemented in large- or medium-sized hospitals because these hospitals usually have more case data and experience, which is conducive to the training and validation of the model, and it can be generalized to smaller hospitals after it passes the clinical practice. The modeling background of the prediction model includes the following: (1) data duration—long-term clinical data, covering relevant case information over the past 10 years, are needed to ensure the stability and accuracy of the model. (2) Data characteristics—the data come from multiple sources, including medical records, imaging studies, and laboratory tests, reflecting the multiple and complex factors affecting recurrence. (3) Modeling purpose—to improve the accuracy of recurrence prediction through ML, help physicians develop more personalized treatment plans, and optimize the allocation of hospital resources. In summary, the clinical environment of the target prediction model is mainly set in the neurosurgical wards of large- or medium-sized hospitals, and the modeling background involves long-term clinical data collection and analysis, aiming to improve prediction accuracy and optimize the allocation of medical resources.

## Results

### Overview

**Figure 4:**
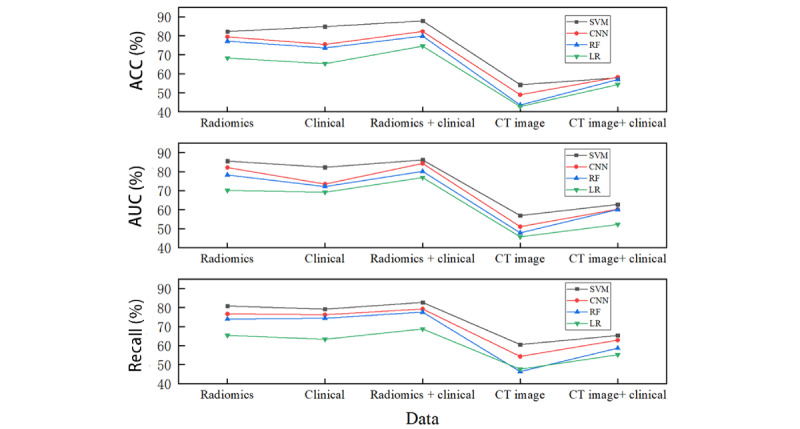
Classification results of different data sets in 4 machine learning models. ACC: accuracy; AUC: area under the curve; CNN: convolutional neural network; CT: computed tomography; LR: linear regression; RF: random forest; SVM: support vector machine.

The primary objective of this study was to streamline the clinical application of the predictive model, specifically by enabling direct input of cranial CT images for generating predictive outcomes. Contrary to expectations, models using CT images as the sole input data type demonstrated suboptimal performance across all evaluation metrics, irrespective of the ML algorithm used. Moreover, models incorporating both clinical data and CT images as input data yielded prediction outcomes significantly inferior to those relying solely on clinical data. Upon using 3D Slicer for radiological feature extraction from the CT images, the resultant model performance exhibited notable improvement. This underscores the inadequacy of current ML algorithms in directly using CT images for clinical research; while predictive results can be generated, they remain unsatisfactory (Figure 4 and Table 2). Consequently, the study abandoned the notion of using image-based input data. In contrast, radiomics data are already a distillation and abstraction of the CT image information, and using the original image as input again may lead to information redundancy and even introduce noise, thus affecting the performance of the model. From the perspective of computational efficiency, direct processing of raw CT images usually requires more computational resources and time, whereas radiomics data, as a more compact and higher-level feature representation, can significantly improve the training and prediction speed of the model. Therefore, in this study, we did not choose to try radiomics data+CT images as input data for model validation. Instead, an exploration into the viability of using clinical data, radiomic features, or a combination thereof as input data sets was conducted to optimize predictive model performance.

**Table 2:** Classification results of different data sets in 4 machine learning models.

Model		Radiomics	Clinical	Clinical-radiomics	CT^a^ image	CT image-clinical
**SVM^b^**						
	Accuracy (%)	82.19	84.82	87.79	54.27	57.86
	AUC^c^ (%)	85.57	82.29	86.13	56.91	62.75
	Recall (%)	80.82	79.16	82.70	60.54	65.32
**CNN^d^**						
	Accuracy (%)	79.42	75.44	82.27	48.95	58.19
	AUC (%)	82.16	73.45	84.32	51.07	60.24
	Recall (%)	76.64	76.18	79.23	54.29	62.85
**RF^e^**						
	Accuracy (%)	77.13	73.58	79.85	43.55	56.97
	AUC (%)	78.27	72.16	80.13	47.82	60.11
	Recall (%)	73.89	74.37	77.62	46.31	58.63
**LR^f^**						
	Accuracy (%)	68.32	65.32	74.56	42.75	54.32
	AUC (%)	70.13	69.17	76.91	45.68	52.19
	Recall (%)	65.34	63.28	68.76	47.54	55.13

^a^CT: computed tomography.

^b^SVM: support vector machine.

^c^AUC: area under the curve.

^d^CNN: convolutional neural network.

^e^RF: random forest.

^f^LR: linear regression.

### Predictive Model Evaluation

This study uses accuracy, AUC, and recall as evaluative metrics for the predictive models, with corresponding results delineated in Figure 4. Accuracy serves as a direct indicator of the model’s consistency in aligning predictive and actual outcomes. Specifically, accuracy represents the ratio of correctly classified samples to the overall sample pool, offering both intuitive understanding and straightforward implementation. It is principally used to assess the model’s ability to accurately categorize target variables in the predictive outcomes. However, accuracy possesses limitations as it exclusively considers the classification of positive samples, thereby omitting negative samples. This lack of comprehensiveness limits accuracy’s capacity to measure the overlap between predicted and true outcomes. To address this limitation, AUC is incorporated as it holistically considers both positive and negative samples, thereby providing a more nuanced evaluation of model performance. Recall, another metric used, is particularly pertinent given the study’s objective to predict the recurrence of CSDH. Recall quantifies the model’s proficiency in accurately identifying positive samples, focusing on true positive cases. It is especially vital for this study, as it emphasizes the model’s ability to correctly predict patient recurrence. Distinct from accuracy and AUC, recall remains unaffected by the selection of a decision threshold, rendering it more appropriate for comparing various models, particularly when the decision threshold is ambiguous or challenging to ascertain. In summary, accuracy, AUC, and recall are deployed as multifaceted evaluative metrics to assess the predictive models constructed in this study.

In a comprehensive evaluation across all designated metrics— accuracy, AUC, and recall—the SVM model consistently outperformed the CNN, RF, and LR models. This was observed irrespective of the input data set used, be it clinical data, radiomics features, or a hybrid of both. For models using radiomics features, SVM demonstrated improvements of 2.77%, 5.06%, and 13.87% in accuracy; 3.41%, 7.3%, and 15.37% in AUC; and 4.18%, 6.93%, and 15.48% in recall compared to CNN, RF, and LR models, respectively. Similarly, when clinical data served as the input, SVM enhanced accuracy by 9.38%, 11.24%, and 19.5%; AUC by 8.84%, 10.13%, and 13.12%; and recall by 2.98%, 4.79%, and 15.88% in comparison to CNN, RF, and LR models, respectively. Interestingly, a combination of clinical and radiomics features as input to the SVM model resulted in further performance gains: accuracy improved by 5.52%, 7.94%, and 13.23%; AUC by 1.81%, 6%, and 9.22%; and recall by 3.47%, 5.08%, and 13.94% in comparison to CNN, RF, and LR models, respectively. These outcomes substantiate the efficacy of the SVM model in predicting postoperative recurrence in patients with CSDH. Moreover, it was observed that the hybrid input set comprising both clinical and radiomics data enhanced the performance of the SVM model itself. Specifically, the accuracy, AUC, and recall were higher by 5.6% and 2.97%; 0.56% and 3.84%; and 1.88% and 3.54%, respectively, when compared to SVM models that used either radiomics features or clinical data as stand-alone inputs. In conclusion, the SVM model, when constructed based on a fusion of clinical and radiomics features, exhibited superior predictive capabilities, making it the optimal choice for this study.

While the SVM model using clinical-radiomics features demonstrated superior performance, it did not meet our predefined target of exceeding 90% across key evaluation metrics. To address this, a feature selection process was implemented to refine the input variables for the predictive model. Our analysis identified the top 5 influential variables impacting postoperative recurrence in patients with CSDH as history of head trauma, MGS, postoperative midline improvement, preoperative midline shift, and radiomics features (Multimedia Appendix 2). History of head trauma is one of the main factors leading to the formation of CSDH; MGS is used to assess the severity of CSDH, including hematoma volume, midline shift, state of consciousness, and other factors; postoperative midline improvement is an important index for assessing surgical results and patient’s recovery; preoperative midline shift reflects the degree of compression of hematoma on brain tissue, if postoperative midline improvement is poor or larger midline shift usually indicates a poor prognosis and a higher risk of recurrence; and radiomics features can provide detailed information about CSDH, which can help physicians more accurately assess the disease and predict the risk of recurrence. These features are valuable in predicting the risk of recurrence after CSDH and should be emphasized by clinicians during diagnosis.

**Figure 5:**
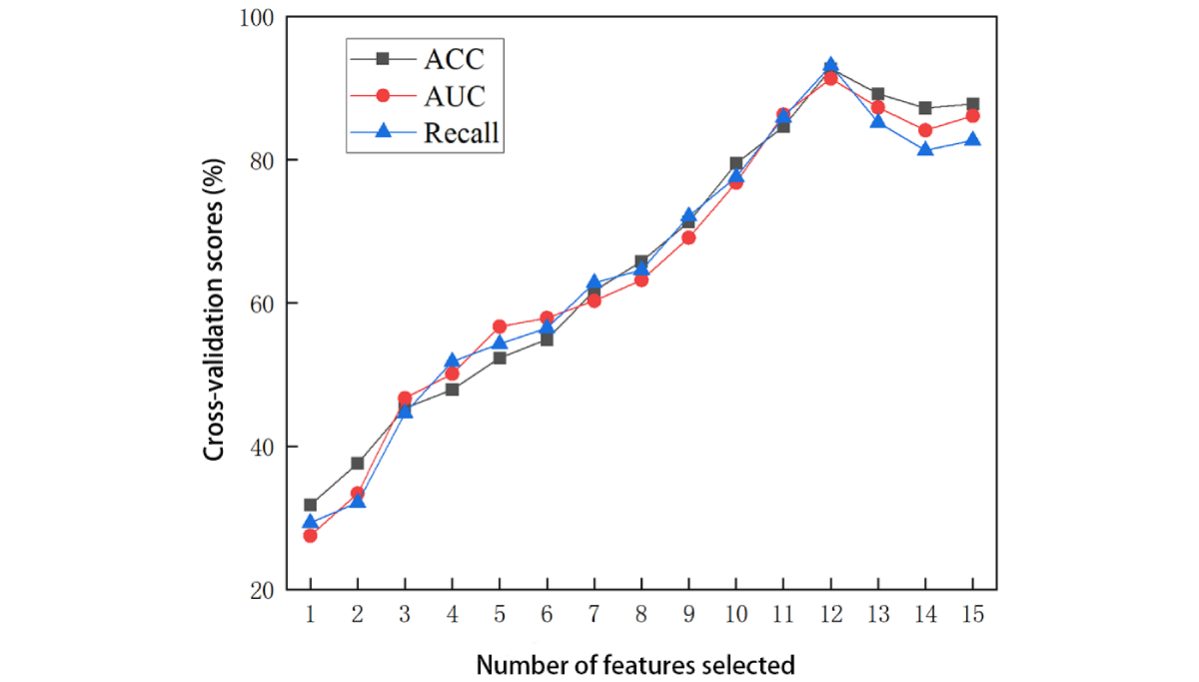
Results of accuracy (ACC), area under the curve (AUC), and recall after selecting the number of features by cross-validation.

Subsequent feature selection experiments, conducted using the RFE method, indicated an optimal combination of 12 variables (Figure 5). When variables such as length of stay in hospital, sex, and age were excluded, the model yielded the highest performance across accuracy, AUC, and recall, registering 92.72%, 91.34%, and 93.16%, respectively. Consequently, these 12 features were incorporated into the refined SVM model to yield an optimal predictive tool for assessing the likelihood of postoperative recurrence in patients with CSDH.

### External Data Set Validation

**Figure 6:**
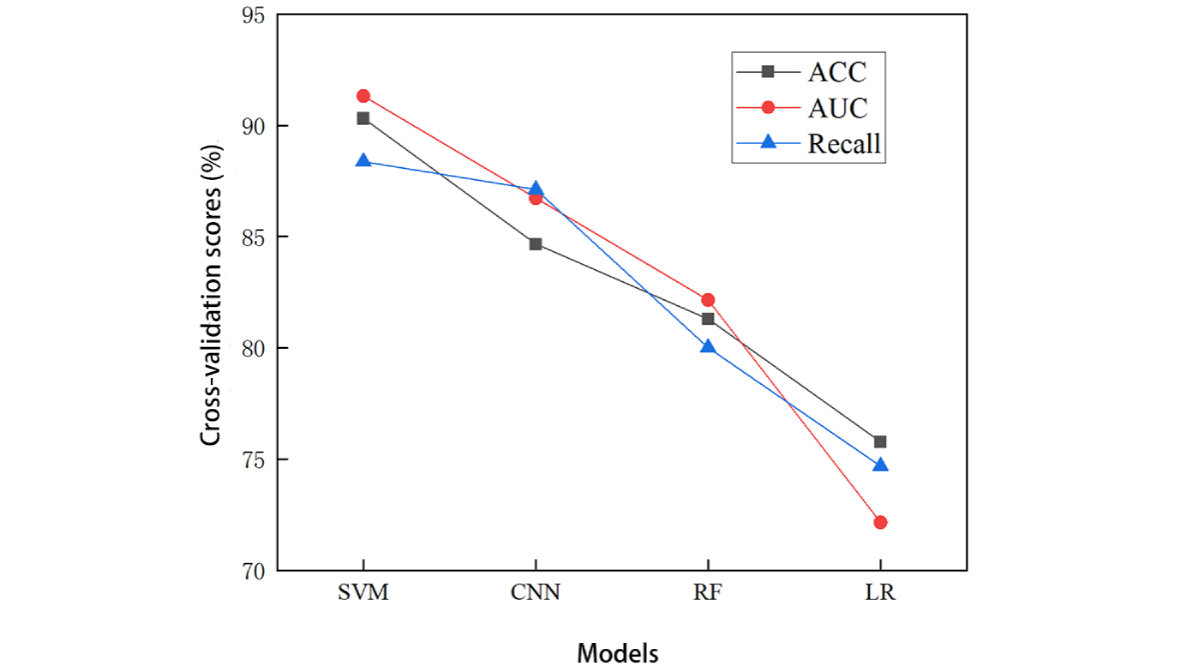
Accuracy (ACC), area under the curve (AUC), and recall for the 4 models in the external validation set. CNN: convolutional neural network; LR: linear regression; RF: random forest; SVM: support vector machine.

To corroborate the reliability and generalizability of the developed SVM model, an external validation was performed using clinical-radiomics feature data from 20 patients with CSDH, sourced from the First Affiliated Hospital of Anhui Medical University. The inclusion criteria, exclusion criteria, outcome metrics, and predictors for the data set used for external validation (including clinical data and preoperative head CT images) were identical to those for the modeling data set (Table 3). There was no significant difference between the distribution of significant variables between the model validation data set and the model development data set when comparing Tables 1 and 3. The obtained data were fed into the 4 preestablished models, and the outcomes are depicted in Figure 6. The accuracy metrics for the 4 models (SVM, CNN, RF, and LR) registered at 90.32%, 84.67%, 81.3%, and 75.78%, respectively. The AUC outcomes were 91.32%, 86.73%, 82.15%, and 72.16%, respectively. Recall rates were recorded at 88.37%, 87.12%, 80.01%, and 74.68%, respectively. Across all evaluation parameters, the SVM model consistently exhibited superior performance. With context, we found that the SVM model constructed based on the fusion of clinical and radiomics features has consistent results in both internal validation and external testing, and performs best among the 4 models. Consequently, these results reconfirm that the SVM model is the most effective predictive tool for assessing postoperative recurrence in patients with CSDH, as further substantiated by this external data set validation.

The data we used for modeling came from the Second Affiliated Hospital of Anhui Medical University, which mainly serves local patients, so its medical record data may reflect more of the disease characteristics and treatment experiences of local patients. The data used for the external validation of the model came from the First Affiliated Hospital of Anhui Medical University, which attracts patients from all over the province and even the neighboring regions due to the hospital’s reputation and geographical location. These patients may have different cultural backgrounds, living habits, and medical needs. Therefore, the 2 aforementioned hospitals provide a context for the differences between the internal and external data sets, ensuring the general applicability of the prediction model.

**Table 3:** Clinical variables used to validate the model.

Variables		Nonrecurrence (n=17)	Recurrence (n=3)	*P* value	
Age (y), mean (SD)		72.3 (10.6)	68.0 (9.6)	.52	
**Sex** **, n (%)**					.53
	Male	14 (82)	3 (100)		
	Female	3 (18)	0 (0)		
**Smoking or drinking, n (%)**					.89
	Yes	12 (71)	2 (67)		
	No	5 (29)	1 (33)		
**Hypertension, n (%)**					.72
	Yes	4 (23)	1 (33)		
	No	13 (77)	2 (67)		
**Cerebral infarction, n (%)**					—^a^
	Yes	0 (0)	0 (0)		
	No	17 (100)	3 (100)		
**History of head trauma, n (%)**					.95
	Yes	10 (59)	2 (67)		
	No	7 (41)	1 (33)		
**History of antiplatelet or anticoagulation** **, n (%)**					.33
	Yes	2 (11.8)	1 (33.3)		
	No	15 (88.2)	2 (66.7)		
**Markwalder Grading Scale, n (%)**					.31
	0	0 (0)	0 (0)		
	1	5 (29)	0 (0)		
	2	9 (53)	3 (100)		
	3	3 (18)	0 (0)		
	4	0 (0.0)	0 (0)		
Length of stay in hospital (d), mean (SD)		14.8 (5.7)	11.7 (3.8)	.38	
**CSDH^b^ location, n (%)**					.58
	Unilateral	13 (77)	3 (100)		
	Bilateral	4 (23)	0 (0)		
**Classification of hematoma, n (%)**					.11
	Homogeneous	4 (23)	0 (0.0)		
	Laminar	3 (18)	0 (0.0)		
	Separated	6 (35)	1 (33)		
	Trabecular type	4 (23)	2 (67)		
Preoperative hematoma volume (mL), mean (SD)		105.7 (27.3)	126.2 (14.6)	.23	
Preoperative midline shift (cm), mean (SD)		1.1 (0.4)	1.3 (0.2)	.39	
Postoperative midline improvement (cm), mean (SD)		0.5 (0.3)	0.6 (0.3)	.59	

^a^Not available.

^b^CSDH: chronic subdural hematoma.

## Discussion

### Principal Findings

In this study, the observed postoperative recurrence rate for patients with CSDH was 14.3%, a figure that aligns closely with previously published data, ranging from 5% to 26% [22,23]. This congruence lends credibility to the representativeness of our data set and suggests that the study’s findings have broad applicability.

Within the neurosurgical landscape, CSDH has received limited investigative focus. This is likely due to its relatively high prevalence and standardized treatment approach, coupled with its lower mortality risk compared to other neurosurgical conditions. However, given the global demographic shift toward an older population, the incidence of CSDH—and consequently, its recurrence rate—is witnessing a steady uptick. This escalating trend underscores the need for ongoing research attention, a need that remains largely unmet.

Using extensive data collection, processing, and iterative model optimization, we arrived at an SVM model predicated on clinical-radiomics features that exhibited optimal predictive performance. The final model not only met our predetermined efficacy criteria but also carries clinical utility. To our understanding, this constitutes the inaugural effort to leverage clinical-radiomics features in tandem with ML methodologies for the relatively accurate forecasting of postoperative recurrence in patients with CSDH. Thus, this study paves the way for future research, offering a novel paradigm for evaluating surgical outcomes in this patient cohort.

Our review of existing literature reveals that although there are studies incorporating ML with medical imaging (CT or magnetic resonance imaging) for clinical applications, the prevailing approach does not typically leverage raw images for model construction. Instead, researchers use various software tools or techniques to extract radiomics features from these images, upon which subsequent models are built. Consistent with these findings, our empirical results confirm that predictive models constructed directly from unprocessed images perform suboptimally. The limitations are primarily attributable to the inherent difficulty that traditional ML algorithms face in extracting key lesion characteristics—such as location, size, and morphology—from unprocessed CT images. To address these limitations, we used the open-source software, 3D Slicer, which offers an array of algorithms for feature extraction, including edge and corner detection, as well as texture analysis. By segmenting the ROI and extracting features using 3D Slicer, we acquired meaningful mathematical attributes, such as gradient and curvature. This enhanced feature set enabled more effective computerized analysis of lesions and improved predictive outcomes. Consequently, we shifted our strategy from using raw CT images to combining extracted radiomics features with clinical data for patients with CSDH as input variables in our model construction. The comparative evaluation of models developed through 4 ML algorithms (SVM, CNN, RF, and LR) revealed the superiority of the hybrid data approach over models built solely on clinical or radiomics data. Further, a performance matrix based on metrics such as accuracy, AUC, and recall indicated marked variations among the 4 methods, with the SVM model outperforming CNN, RF, and LR on all 3 metrics (accuracy: 2.77%, 5.06%, and 13.87%; AUC: 3.41%, 7.3%, and 15.44%; and recall: 4.18%, 6.93%, and 15.48%, respectively). Therefore, our analysis corroborates the high reliability of the SVM-based predictive model constructed using the amalgamated data set.

Our analysis of the reasons for the poor performance of other models is as follows. (1) Mismatch between data characteristics and model complexity: some highly complex models, such as CNN for deep learning, may encounter overfitting problems when confronted with data sets that are relatively small or not rich enough in feature dimensions; this means that the model performs well on training data, but performs poorly on new, unseen data. (2) Feature extraction and exploitation capabilities: while RF and LR have some advantages in dealing with nonlinear problems and feature selection, they may not be as good as especially designed algorithms such as SVM with appropriate kernel functions. (3) Sensitivity to unbalanced data: models such as RF and LR may be biased toward the majority class if the positive and negative samples are unevenly distributed, which is a common problem in medical image analysis. This may lead to a decrease in the recall of the model and weak identification of the minority class (recurrent cases in this study). And the main reason why SVM performs well in predicting postoperative recurrence in patients with CSDH is that its principle of maximum interval (SVM improves the generalization ability of the model by maximizing the interval between the decision boundary and the support vectors) and the kernel trick (SVM can map the input space to a high-dimensional feature space through the kernel function, in which the nonlinear problem may become linearly differentiable) provide powerful support for dealing with small samples, high dimensionality, and unbalanced medical data provide powerful support.

Our model achieved 90.32% accuracy, 91.32% AUC, and 88.37% recall on an independent external validation set. These metrics indicate that the model has high accuracy in predicting postoperative recurrence in patients with CSDH. For socioeconomic purposes, accurate prediction can reduce unnecessary examinations and follow-up visits, which can save a large amount of medical resources every year; accurate prediction can also help physicians to take interventions in advance to reduce the incidence of postoperative complications and readmission rate of patients. This not only reduces patient suffering but also lowers hospital readmission costs. For patients, it reduces their burden by decreasing unnecessary examinations and follow-up visits, and timely interventions based on the predicted results can help reduce the occurrence of complications and improve their quality of life; in addition, through the model prediction, physicians can provide patients with more personalized treatment plans and care recommendations, which can improve patients’ satisfaction and trust. In summary, our model has excellent performance and significant impact on clinical practice and economic benefits.

As early as 2009, Abouzari et al [24] explored the use of ML algorithms, specifically artificial neural networks and LR, for prognosis prediction in patients with CSDH. Given the technological limitations of that era, these models exhibited low accuracy and questionable evaluation metrics. However, their pioneering work served as a catalyst for us to implement contemporary ML techniques in this research domain.

With respect to postoperative recurrence in patients with CSDH, extensive studies have been conducted on hematoma staging using CT imaging. Historically, hematomas were simplistically categorized into 4 density-based types: low density, isodense, high density, and mixed density [25]. However, Tsutsumi et al [26] found no statistically significant difference in postoperative recurrence rates when using these classifications. Subsequently, Nakaguchi et al [17] introduced an alternative, more nuanced, classification—comprising homogeneous, laminar, separated, and trabecular types—which garnered wide acceptance in the research community.

In the current investigation, we adhered to this latter classification scheme when analyzing CT images. Notably, our data analysis revealed that the “separated” type constituted a greater fraction of the recurrence group, aligning with prior research findings. However, the proportion of cases classified as “trabecular” diverged from existing literature. We hypothesize that this discrepancy may be attributable to selection bias arising from our limited data set.

In the realm of CSDH postoperative recurrence, the scholarly focus has predominantly been on surgical methodologies—recently emphasizing middle meningeal artery embolization—patient age, and the administration of antiplatelet or anticoagulant medications [27-29]. This narrow concentration likely stems from the ubiquity of CSDH and the established efficacy of existing surgical treatments, which generally yield favorable outcomes without posing immediate life-threatening risks to patients. Consequently, research has largely remained at the clinical echelon. However, as technological advancements continue to pervade medical practice, the incorporation of these innovations not only streamlines clinical operations but also enhances patient outcomes, thereby advancing the objective of precision medicine.

In the current investigation, we diverged from the conventional practice of using either clinical data or imaging histology data exclusively. Rather, we integrated both data types and, through comparative analysis, substantiated the superior predictive performance of combined clinical-radiomics features. Furthermore, SVM was identified as an efficacious classification algorithm particularly suited for the unique characteristics of medical imaging data, which are high-dimensional and often limited in sample size. SVM achieves classification by constructing a hyperplane that appropriately segregates distinct feature sets in medical imaging data, thereby facilitating more accurate identification and prediction of postoperative recurrence in patients with CSDH. In addition, SVM exhibits robustness in mitigating the influence of noise and outliers commonly present in radiomics features, thus bolstering the reliability of model predictions.

In the realm of medical research, feature selection predominantly uses filtering methods, including but not limited to correlation coefficients, chi-square tests, and mutual information, to identify variables that highly correlate with the target outcome. Filtering methods excel in computational efficiency, capable of swiftly processing large data sets and thereby reducing dimensionality. These methods also offer adaptability, accommodating user-defined criteria for application-specific scenarios.

Nevertheless, this study uses RFE in lieu of filtering methods, and for several substantive reasons: Capability to manage highly correlated features—filtering methods struggle with the presence of a multitude of highly correlated variables, a challenge more effectively navigated by RFE. Distributional assumption sensitivity—filtering methods often rest on certain statistical distribution assumptions (eg, normal, t-distribution), which, if incorrect, compromise feature selection accuracy. Conversely, RFE operates independently of such assumptions. Computational efficiency—contrary to common perception, filtering methods, while efficient with smaller data sets, may demand substantial computational resources and time when applied to larger data sets. RFE, on the other hand, demonstrates superior computational efficiency in such contexts. Applicability to nonlinear problems—filtering methods generally rely on linear models, limiting their efficacy for nonlinear challenges. RFE exhibits no such constraint. Automated and robust feature selection—unique to RFE is its ability to automatically discern the most pertinent feature subset, obviating the need for manual selection. This automation further minimizes overfitting risks and enhances model interpretability by focusing on the most salient features [30,31].

Given these advantages, RFE was selected as the feature selection methodology for this study.

Upon implementing RFE for feature selection, the predictive model demonstrated robust performance metrics, including an accuracy of 92.72%, AUC of 91.34%, and recall rate of 93.16%. Experimental outcomes identified the top 5 variables influencing postoperative recurrence in patients with CSDH as follows: history of head trauma, MGS, postoperative midline improvement, preoperative midline shift, and radiomics. Notably, the substantial impact of postoperative midline improvement and preoperative midline shift on the prognosis of CSDH has not been highlighted in extant literature. Consequently, we advocate for the inclusion of these novel factors in future CSDH studies given their potential clinical relevance.

In the clinical setting, the specific steps we take to implement the model prediction function are as follows: (1) integrate the predictive model into an existing health care information system, such as an electronic medical record system or medical image processing software; (2) input data into the system, which consists of the patient’s medical images and relevant clinical information; (3) predictive results are presented to the physician in an easy-to-understand manner, such as a percentage of probability representation; and (4) regularly update and maintain the prediction model. This can be done by collecting new clinical data, optimizing algorithm parameters, or adjusting feature selection strategies. Through the abovementioned steps, patients can be provided with personalized treatment plans, reducing unnecessary tests and surgeries and improving the efficient use of medical resources. For patients with a higher risk of recurrence, physicians can take treatment measures earlier, thus improving the patient’s prognosis. In the event of a discrepancy between the model prediction and the physician’s judgment, the physician should first adopt a conservative treatment strategy to ensure patient safety. For example, patients whose model predictions are at low risk of recurrence but whom physicians believe are at higher risk should continue to be closely monitored and followed up. The physician can then compare the model predictions with the patient’s actual treatment results and feed this information back to the model developer. Through continuous data feedback and model optimization, the predictive accuracy and generalization ability of the model can be improved. For now, ML models are only supplementary tools, and physicians should always make the final decision in conjunction with their own expertise and experience.

To realize the effective application of predictive models in clinical practice, we need to establish a stable and reliable data pipeline. First, we need to collect clinical data from patients with CSDH, including radiomic features, medical record information, and surgical records. Then, we need to preprocess and feature extract the data to feed it into a predictive model. Next, we need to train and validate the model using ML algorithms to ensure its predictive accuracy and reliability. Finally, we need to integrate the predictive model into an existing health care information system so that it can automatically receive and process patients’ clinical data and generate predictions. During the establishment of the data pipeline, we need to consider the quality, integrity, and security of the data. We need to ensure the accuracy and consistency of the data to avoid adverse effects on the predicted outcomes. At the same time, we need to ensure data security and privacy to protect patients’ privacy rights.

While our findings hold considerable clinical utility and prospective applicability, it is imperative to acknowledge the following limitations of the study: Assumed input and output data formats—the ML model used in this study is based on a specific input data format (eg, radiological features and clinical data extracted via 3D Slicer software) and assumes that these data are fully representative of the patient’s health status and subdural hematoma characteristics. However, this assumption may omit other important biomarkers or unquantified clinical parameters [19,20], which may have an impact on the predictive power of the model. The output data format is assumed to be measured in terms of specific predictive accuracy metrics (eg, accuracy, AUC, and recall), which may not adequately reflect the utility and sensitivity of the model in different clinical settings. Potential pitfalls in interpreting the model—although the SVM model showed good performance in this study, SVMs and other ML models are often considered “black-box” models, in which the model’s decision-making process may not be transparent, and this lack of interpretability may produce a lack of trust in settings where the model is used to guide clinical decision-making. Potential bias of the data used in modeling—the study was conducted based on a retrospective data set from a specific health care organization, which may be subject to selection bias (eg, only patients who received surgical treatments were included) and informational bias (data records may not be completely accurate). In addition, due to the relatively small sample size (133 patients), the complex relationship between CSDH recurrence and multiple underlying factors may not have been adequately captured, which may have affected the model’s ability to generalize and predict accuracy. Generalizability of the data—although the study was externally validated, the validation set consisted of only 20 patients from another health care facility, which may not be sufficient to comprehensively assess the ability of the model to generalize across populations and geographical regions. Patient populations in different regions may have different clinical characteristics, such as different treatment modalities and different health care resources, all of which may affect the generalizability and accuracy of the model.

Our subsequent research will work to address the above issues.

### Conclusions

In this study, we constructed 4 models to predict postoperative recurrence in patients with CSDH, using ML algorithms and an amalgamated data set comprising both radiomics attributes and clinical variables. Comparative evaluation revealed that the SVM model, using this integrated data set, demonstrated superior predictive accuracy. The model not only outperforms previously established methods but also provides a more specific and comprehensive framework for predicting outcomes. These predictive findings enable health care teams to refine clinical decision-making and offer individualized treatment plans. Moreover, patients can engage in proactive follow-up and informed participation in their treatment protocols based on these results. The developed method offers the advantage of real-time updates and holds considerable clinical implications.

## Appendix

Multimedia Appendix 1 Items of reporting predictive models in biomedical research.

Multimedia Appendix 2 Ranking the importance of these 12 features screened by the recursive feature elimination method in descending order of importance. CSDH: chronic subdural hematoma.

## Abbreviations

AUC: area under the curve
CNN: convolutional neural network
CSDH: chronic subdural hematoma
CT: computed tomography
LR: linear regression
MGS: Markwalder Grading Scale
ML: machine learning
RF: random forest
RFE: recursive feature elimination
ROI: region of interest
SVM: support vector machine

## References

[1] Kudo H, Kuwamura K, Izawa I, Sawa H, Tamaki N (1992). Chronic subdural hematoma in elderly people: present status on Awaji Island and epidemiological prospect. Neurol Med Chir (Tokyo).

[2] Rust T, Kiemer N, Erasmus A (2006). Chronic subdural haematomas and anticoagulation or anti-thrombotic therapy. J Clin Neurosci.

[3] Feghali J, Yang W, Huang J (2020). Updates in chronic subdural hematoma: epidemiology, etiology, pathogenesis, treatment, and outcome. World Neurosurg.

[4] Deo RC (2015). Machine learning in medicine. Circulation.

[5] Li C, Liu M, Zhang Y, Wang Y, Li J, Sun S, Liu X, Wu H, Feng C, Yao P, Jia Y, Zhang Y, Wei X, Wu F, Du C, Zhao X, Zhang S, Qu J (2023). Novel models by machine learning to predict prognosis of breast cancer brain metastases. J Transl Med.

[6] Wang X, Zhong J, Lei T, Chen D, Wang H, Zhu L, Chu S, Liu L (2021). An artificial neural network prediction model for posttraumatic epilepsy: retrospective cohort study. J Med Internet Res.

[7] Yao Q, Jia W, Chen S, Wang Q, Liu Z, Liu D, Ji X (2023). Machine learning was used to predict risk factors for distant metastasis of pancreatic cancer and prognosis analysis. J Cancer Res Clin Oncol.

[8] Monteiro M, Newcombe VF, Mathieu F, Adatia K, Kamnitsas K, Ferrante E, Das T, Whitehouse D, Rueckert D, Menon DK, Glocker B (2020). Multiclass semantic segmentation and quantification of traumatic brain injury lesions on head CT using deep learning: an algorithm development and multicentre validation study. Lancet Digit Health.

[9] Fang C, Pan Y, Zhao L, Niu Z, Guo Q, Zhao B (2022). A machine learning-based approach to predict prognosis and length of hospital stay in adults and children with traumatic brain injury: retrospective cohort study. J Med Internet Res.

[10] Gillies RJ, Kinahan PE, Hricak H (2016). Radiomics: images are more than pictures, they are data. Radiology.

[11] Lambin P, Leijenaar RT, Deist TM, Peerlings J, de Jong EE, van Timmeren J, Sanduleanu S, Larue RT, Even AJ, Jochems A, van Wijk Y, Woodruff H, van Soest J, Lustberg T, Roelofs E, van Elmpt W, Dekker A, Mottaghy FM, Wildberger JE, Walsh S (2017). Radiomics: the bridge between medical imaging and personalized medicine. Nat Rev Clin Oncol.

[12] Vallières M, Zwanenburg A, Badic B, Cheze Le Rest C, Visvikis D, Hatt M (2018). Responsible radiomics research for faster clinical translation. J Nucl Med.

[13] Esteva A, Kuprel B, Novoa RA, Ko J, Swetter SM, Blau HM, Thrun S (2017). Dermatologist-level classification of skin cancer with deep neural networks. Nature.

[14] Schwier M, van Griethuysen J, Vangel MG, Pieper S, Peled S, Tempany C, Aerts HJ, Kikinis R, Fennessy FM, Fedorov A (2019). Repeatability of multiparametric prostate MRI radiomics features. Sci Rep.

[15] Zhao B, Tan Y, Tsai WY, Qi J, Xie C, Lu L, Schwartz LH (2016). Reproducibility of radiomics for deciphering tumor phenotype with imaging. Sci Rep.

[16] Markwalder TM (1981). Chronic subdural hematomas: a review. J Neurosurg.

[17] Nakaguchi H, Tanishima T, Yoshimasu N (2001). Factors in the natural history of chronic subdural hematomas that influence their postoperative recurrence. J Neurosurg.

[18] Stanišic M, Pripp AH (2017). A reliable grading system for prediction of chronic subdural hematoma recurrence requiring reoperation after initial burr-hole surgery. Neurosurgery.

[19] Hirai S, Yagi K, Hara K, Kanda E, Matsubara S, Uno M (2021). Postoperative recurrence of chronic subdural hematoma is more frequent in patients with blood type A. J Neurosurg.

[20] Matsubara M, Yagi K, Minami Y, Kanda E, Sunada Y, Tao Y, Takai H, Shikata E, Hirai S, Matsubara S, Uno M (2023). Preoperative elevated eosinophils in peripheral blood for prediction of postoperative recurrence of chronic subdural hematoma. J Neurosurg.

[21] Pan Y, Fang C, Zhu X, Wan J (2023). Construction of a predictive model based on MIV-SVR for prognosis and length of stay in patients with traumatic brain injury: retrospective cohort study. Digit Health.

[22] Martinez-Perez R, Tsimpas A, Rayo N, Cepeda S, Lagares A (2021). Role of the patient comorbidity in the recurrence of chronic subdural hematomas. Neurosurg Rev.

[23] Weigel R, Schmiedek P, Krauss JK (2003). Outcome of contemporary surgery for chronic subdural haematoma: evidence based review. J Neurol Neurosurg Psychiatry.

[24] Abouzari M, Rashidi A, Zandi-Toghani M, Behzadi M, Asadollahi M (2009). Chronic subdural hematoma outcome prediction using logistic regression and an artificial neural network. Neurosurg Rev.

[25] Nomura S, Kashiwagi S, Fujisawa H, Ito H, Nakamura K (1994). Characterization of local hyperfibrinolysis in chronic subdural hematomas by SDS-PAGE and immunoblot. J Neurosurg.

[26] Tsutsumi K, Maeda K, Iijima A, Usui M, Okada Y, Kirino T (1997). The relationship of preoperative magnetic resonance imaging findings and closed system drainage in the recurrence of chronic subdural hematoma. J Neurosurg.

[27] Cofano F, Pesce A, Vercelli G, Mammi M, Massara A, Minardi M, Palmieri M, D'Andrea G, Fronda C, Lanotte MM, Tartara F, Zenga F, Frati A, Garbossa D (2020). Risk of recurrence of chronic subdural hematomas after surgery: a multicenter observational cohort study. Front Neurol.

[28] Shotar E, Meyblum L, Premat K, Lenck S, Degos V, Grand T, Cortese J, Pouvelle A, Pouliquen G, Mouyal S, Boch AL, Carpentier A, Sourour NA, Mathon B, Clarençon F (2020). Middle meningeal artery embolization reduces the post-operative recurrence rate of at-risk chronic subdural hematoma. J Neurointerv Surg.

[29] Andersen-Ranberg NC, Poulsen FR, Bergholt B, Hundsholt T, Fugleholm K (2017). Bilateral chronic subdural hematoma: unilateral or bilateral drainage?. J Neurosurg.

[30] Yan K, Zhang D (2015). Feature selection and analysis on correlated gas sensor data with recursive feature elimination. Sens Actuators B Chem.

[31] Chen XW, Jeong JC (2007). Enhanced recursive feature elimination. Proceedings of the 6th International Conference on Machine Learning and Applications.

