# Supplementary material for: Combining Clinical-Radiomics Features With Machine Learning Methods for Building Models to Predict Postoperative Recurrence in Patients With Chronic Subdural Hematoma: Retrospective Cohort Study
Source: J Med Internet Res. 2024 Aug 28;26:e54944. doi: 10.2196/54944 (PMC11391156; doi:10.2196/54944)
Supplement: Multimedia Appendix 1 [file jmir_v26i1e54944_app1.docx]

**Appendix**

Items of reporting predictive models in biomedical research.

| Item number | Section | Topic | Checklist item | Is this entry included |
| --- | --- | --- | --- | --- |
| 1 | Title | Nature of study | Identify the report as introducing a predictive model. | Yes |
| 2 | Abstract | Structured summary | Background. | Yes |
|  |  |  | Objectives. | Yes |
|  |  |  | Data sources. | Yes |
|  |  |  | Performance metrics of the predictive model or models. | Yes |
|  |  |  | Conclusion including the practical value of the developed predictive model or models. | Yes |
| 3 | Introduction | Rationale | Identify the clinical goal. | Yes |
|  |  |  | Review the current practice and prediction accuracy of any existing models. | Yes |
| 4 |  | Objectives | State the nature of study being predictive modeling, defining the target of prediction. | Yes |
|  |  |  | Identify how the prediction problem may benefit the clinical goal. | Yes |
| 5 | Methods | Describe the setting | Identify the clinical setting for the target predictive model. | Yes |
|  |  |  | Identify the modeling context in terms of facility type, size, volume, and duration of available data. | Yes |
| 6 |  | Define the prediction problem | Define a measurement for the prediction goal. | Yes |
|  |  |  | Determine that the study is retrospective or prospective. | Yes |
|  |  |  | Identify the problem to be prognostic or diagnostic. | Yes |
|  |  |  | Determine the form of the prediction model: (1) classification if the target variable is categorical, (2) regression if the target variable is continuous, (3) survival prediction if the target variable is the time to an event. | Yes |
|  |  |  | Translate survival prediction into a regression problem, with the target measured over a temporal window following the time of prediction. | No |
|  |  |  | Explain practical costs of prediction errors. | No |
|  |  |  | Defining quality metrics for prediction models. | Yes |
|  |  |  | Define the success criteria for prediction. | Yes |
| 7 |  | Prepare data for model building | Identify relevant data sources and quote the ethics approval number for data access. | Yes |
|  |  |  | State the inclusion and exclusion criteria for data. | Yes |
|  |  |  | Describe the time span of data and the sample or cohort size. | Yes |
|  |  |  | Define the observational units on which the response variable and predictor variables are defined. | Yes |
|  |  |  | Define the predictor variables. Extra caution is needed to prevent information leakage from the response variable to predictor variables. | Yes |
|  |  |  | Describe the data preprocessing performed, including data cleaning and transformation. | Yes |
|  |  |  | Remove outliers with impossible or extreme responses; state any criteria used for outlier removal. | Yes |
|  |  |  | State how missing values were handled. | Yes |
|  |  |  | Describe the basic statistics of the dataset, particularly of the response variable. These include the ratio of positive to negative classes for a classification problem and the distribution of the response variable for regression problem. | Yes |
|  |  |  | Define the model validation strategies. | Yes |
|  |  |  | Internal validation is the minimum requirement; external validation should also be performed whenever possible. | Yes |
|  |  |  | Specify the internal validation strategy. | Yes |
|  |  |  | Common methods include random split, time-based split, and patient-based split. | Yes |
|  |  |  | Define the validation metrics. For regression problems, the normalized root-mean-square error should be used. For classification problems, the metrics should include sensitivity, specificity, positive predictive value, negative predictive value, area under the ROC curve, and calibration plot | Yes |
|  |  |  | For retrospective studies, split the data into a derivation set and a validation set. For prospective studies, define the starting time for validation data collection. | Yes |
| 8 |  | Build the predictive model | Identify independent variables that predominantly take a single value. | Yes |
|  |  |  | Identify and remove redundant independent variables. | Yes |
|  |  |  | Identify the independent variables that may suffer from the perfect separation problem. | Yes |
|  |  |  | Report the number of independent variables, the number of positive examples, and the number of negative examples. | Yes |
|  |  |  | Assess whether sufficient data are available for a good fit of the model. In particular, for classification, there should be a sufficient number of observations in both positive and negative classes. | Yes |
|  |  |  | Determine a set of candidate modeling techniques (eg, logistic regression, random forest, or deep learning). If only one type of model was used, justify the decision for using that model. | Yes |
|  |  |  | Define the performance metrics to select the best model. | Yes |
|  |  |  | Specify the model selection strategy. | Yes |
|  |  |  | Common methods include K-fold validation or bootstrap to estimate the lost function on a grid of candidate parameter values. For K-fold validation, proper stratification by the response variable is needed. | Yes |
|  |  |  | For model selection, include discussion on (1) balance between model accuracy and model simplicity or interpretability, and (2) the familiarity with the modeling techniques of the end user. | Yes |
| 9 | Results | Report the final model and performance | Report the predictive performance of the final model in terms of the validation metrics specified in the methods section. | Yes |
|  |  |  | If possible, report the parameter estimates in the model and their confidence intervals. When the direct calculation of confidence intervals is not possible, report nonparametric estimates from bootstrap samples. | No |
|  |  |  | Comparison with other models in the literature should be based on confidence intervals. | No |
|  |  |  | Interpretation of the final model. If possible, report what variables were shown to be predictive of the response variable. State which subpopulation has the best prediction and which subpopulation is most difficult to predict. | Yes |
| 10 | Discussion | Clinical implications | Report the clinical implications derived from the obtained predictive performance. | Yes |
| 11 |  | Limitations of the model | Discuss the following potential limitations: • Assumed input and output data format • Potential pitfalls in interpreting the model^a^ • Potential bias of the data used in modeling • Generalizability of the data | Yes |
| 12 |  | Unexpected results during the experiments | Report unexpected signs of coefficients, indicating collinearity or complex interaction between predictor variables. | No |
