# Supplementary figures and images for: Combining Clinical-Radiomics Features With Machine Learning Methods for Building Models to Predict Postoperative Recurrence in Patients With Chronic Subdural Hematoma: Retrospective Cohort Study
Source: J Med Internet Res. 2024 Aug 28;26:e54944. doi: 10.2196/54944 (PMC11391156; doi:10.2196/54944)

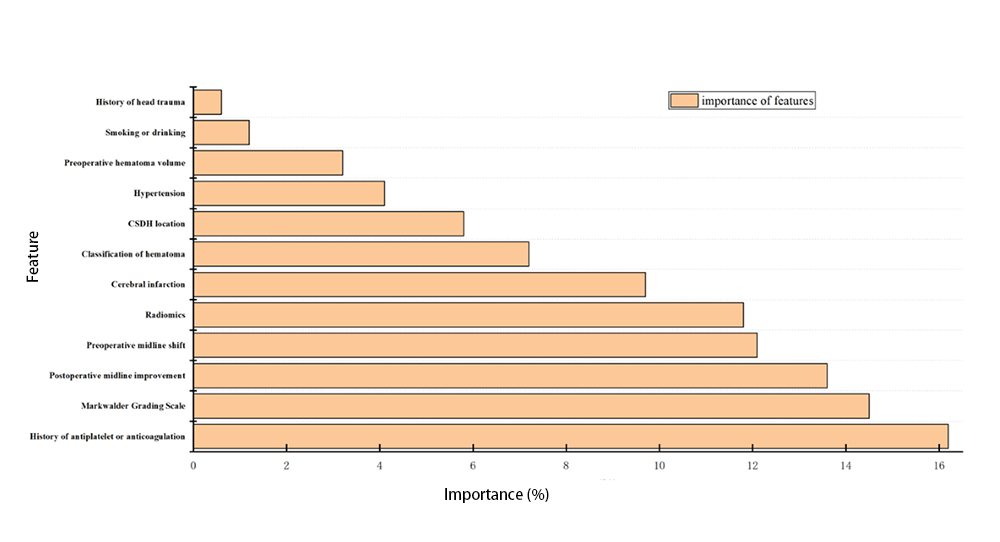

Supplement: Multimedia Appendix 2 [file jmir_v26i1e54944_app2.png]
